# High Resolution Spatial and Temporal Mapping of Traffic-Related Air Pollutants

**DOI:** 10.3390/ijerph120403646

**Published:** 2015-04-01

**Authors:** Stuart Batterman, Rajiv Ganguly, Paul Harbin

**Affiliations:** 1 Environmental Health Sciences, School of Public Health, University of Michigan, Ann Arbor, MI 48109, USA; 2 Department of Civil Engineering, Jaypee University of Information Technology, Waknaghat, Solan, Himachal Pradesh 173234, India; E-Mail: rajiv.ganguly@juit.ac.in; 3 Institute for Population Health, 1400 E. Woodbridge, Detroit, MI 48207, USA; E-Mail: pharbin@ipophealth.org

**Keywords:** air pollution, dispersion models, human exposure, PM_2.5_, traffic

## Abstract

Vehicle traffic is one of the most significant emission sources of air pollutants in urban areas. While the influence of mobile source emissions is felt throughout an urban area, concentrations from mobile emissions can be highest near major roadways. At present, information regarding the spatial and temporal patterns and the share of pollution attributable to traffic-related air pollutants is limited, in part due to concentrations that fall sharply with distance from roadways, as well as the few monitoring sites available in cities. This study uses a newly developed dispersion model (RLINE) and a spatially and temporally resolved emissions inventory to predict hourly PM_2.5_ and NO_x_ concentrations across Detroit (MI, USA) at very high spatial resolution. Results for annual averages and high pollution days show contrasting patterns, the need for spatially resolved analyses, and the limitations of surrogate metrics like proximity or distance to roads. Data requirements, computational and modeling issues are discussed. High resolution pollutant data enable the identification of pollutant “hotspots”, “project-level” analyses of transportation options, development of exposure measures for epidemiology studies, delineation of vulnerable and susceptible populations, policy analyses examining risks and benefits of mitigation options, and the development of sustainability indicators integrating environmental, social, economic and health information.

## 1. Introduction

Vehicle-related emissions can cause serious air pollution problems in many areas, and air pollution associated with traffic is a widespread environmental concern [1] Exposure to traffic generated pollutants, which include oxides of nitrogen (NO_x_), carbon monoxide (CO), volatile organic carbon (VOC) and particulate matter (PM), can cause adverse health effects such as impaired lung function and asthma [2,3], deficits in lung function growth [4], and cancer [5,6]. Vulnerable groups include individuals with existing respiratory and cardiovascular disease, e.g., children with asthma [7,8]. Traffic-related air pollutants show steep gradients in concentrations with distance from major roads [9], so individuals living or working near major roads could have the highest exposures. 

Air pollutant exposures and specifically impacts due to traffic-related emissions can be estimated using a variety of methods, but there are significant gaps and practical issues [10,11,12,13]. In many cities, information regarding the share of pollution attributable to traffic sources and the variation over time is extremely limited. Indicators or surrogate metrics like proximity to roads can be overly simplistic and inadequate since these metrics exclude important factors affecting both emissions (e.g., traffic volume and fleet mix) and dispersion (e.g., meteorology) [12]. While some air pollutants are regularly monitored at several locations in large cities, the number of monitoring locations is never adequate to show the spatial patterns. Hence, various sorts of air quality models can be used to help obtain the spatial and temporal variations of traffic-generated pollutants. These include “dispersion” models using a variety of statistical (e.g., Gaussian plume) and physically-based (e.g., computational fluid dynamic) models that simulate emissions and dispersion [14]; “land use regression” (LUR) models fitting concentrations measured at multiple sites using statistical models and land characteristics, traffic and other data as independent variables, which then are used to predict concentrations elsewhere [15]; “receptor” models using measured pollutant characteristics as tracers to identify and quantify emission sources [16], and eddy-correlation and other methods that evaluate pollutant emissions arising from traffic [17].

The use of geocoded data and geographical information systems (GIS) has become routine in many types of environmental analyses. While surrogates of pollutant exposure have been widely used, e.g., the distance from residences or schools to highways or Superfund sites [18,19], such metrics can have significant limitations: they incompletely or improperly account for the nature of emission sources, effects of meteorology, orographic features, small scale variation in pollutant concentrations, time-activity patterns of emissions and the study subjects, and other factors that can affect pollutant emissions, transport, fate and exposure. In consequence, results may be biased and exposures may be misclassified [12]. In addition, surrogates do not provide quantitative exposure estimates, which restrict interpretations and uses in policy development and management since results cannot be compared to ambient air quality standards. 

The present analysis is motivated by the ‘Near-road EXposures and effects of Urban air pollutants Study’ (NEXUS), which has the objective of investigating the adverse health effects of traffic-related air pollutants in a cohort of asthmatic children living close to major roads in Detroit (Michigan, USA). NEXUS differs from other cohort studies in its use of air quality dispersion modeling to characterize the spatial and temporal variability of traffic-related air pollutants, which is used as an input to determine the exposure estimates [20]. In addition, NEXUS uses a hybrid modeling system that combines several physically-based simulation models to estimate hourly, daily, and long term concentrations, including a new line source dispersion model designed specifically for predicting concentrations from on-road sources, which is featured in the present analysis.

This paper presents an analysis of PM_2.5_ and NO_x_ concentrations that result from traffic emissions at very high spatial and temporal resolution across Detroit, a large urban area. The analysis includes hourly to annual averaging periods and resolutions as fine as 10 m. The paper discusses the development of the modeling system, data and computational issues. After presenting key results, several applications and recommendations for high resolution air quality information are presented. 

## 2. Methodology

Datasets needed to estimate air pollutant concentrations using dispersion models include receptor locations, meteorology, road network, traffic information, and emissions factors. The following sections summarize these datasets and the modeling approach.

### 2.1. Geographic Domain Receptors

The study domain encompasses Detroit and much of surrounding Wayne County in Michigan, USA. Concentrations were calculated at 27,622 “receptors” on 150 m centers over a region 34.5 (E-W) × 23 (N-S) km in dimension, with the SW Universal Transverse Mercator (UTM) coordinates of (311,500, 4,680,500, Region 17). The southeast (SE) corner of the region, over the Detroit River, Lake St. Clair and Canada, is not included. Each receptor represents a discrete point or location, although the prediction for that receptor can represent concentrations over its 150 × 150 m grid cell. The receptor location is the center of the cell. The 150 m spacing was selected to balance the spatial variation expected with computational considerations, as discussed later. We also demonstrate 10 m spacing for a subset of the modeling domain. 

### 2.2. Meteorology

Meteorological data included hourly surface data (e.g., surface wind speed, wind direction, and temperature) from Detroit City airport, which was determined to be representative of the study area. Data for the year 2010 through 2012 were processed by AERMET, which extracted data from data archives, completed quality assessment checks, merged surface, upper air and on-site data, and estimated boundary layer parameters. Unless otherwise stated, analyses reported here use 2010 data. AERMET produces files that contain surface data (e.g., hourly boundary layer parameters); and profile data (e.g., multiple level observations of wind speed, wind direction, temperature and the standard deviation of wind components). 

While meteorological datasets are generally quite complete (typically greater than 95% available and valid data), the lack of complete data can skew dispersion modeling results, and hence it becomes imperative (if possible) to compute predictions based on a full set of meteorological data. Some of the data missing at Detroit City airport were replaced with data from the four surrounding airports in or near to the modeling domain (Detroit Wayne, St. Claire, Gross Isle, Windsor). Measurements at these sites typically showed very close agreement. For invalid or missing wind direction data, replacement values were rotated slightly to improve agreement.

The friction velocity parameter U^*^ was re-calculated if the wind speed changed from the original meteorological file. U^*^ values were computed using the iterative formula in AERMET. Similarly, the Monin-Obhukov (MO) length was calculated using the following equation:

L_MO_ = ρ C TEMP_ref_ U^*3^ / (k g H) (1)

where ρ = density of air = 1.2041 kg/m^3^, C = specific heat capacity of air = 1 kJ/kg, and TEMP_ref_ = the reference temperature for that particular hour; k = von Karman constant = 0.4; g = gravitational constant = 9.81 m/s^2^; H = heat flux = −ρ C U^*^ θ^*^, where θ^*^ was calculated using the expression in AERMET. Finally, the height of mechanically generated boundary layer was computed using U*:

H_BL_ = 2300 U^* 1.5^ (2)

Other parameters missing from the AERMET pre-processor were replaced using standard values. Relatively few (5%) replacements were needed to obtain a complete set of meteorological parameters.

### 2.3. Roadway Links Traffic Activity

Road network data for the Detroit study area, including the coordinates (start/stop locations) of individual links, link classifications, annual average daily traffic (AADT) and average speed information, were provided by the Southeast Michigan Council of Governments [21] for 9701 road links. For the larger roads, e.g., major arterials and interstate highways, each road direction was represented by a separate link. These link data do not include local roads, e.g., neighborhood streets and alleys, but these streets generally have very little traffic.

Hourly traffic volume, fleet mix and vehicle speed was estimated for each link, information used to estimate emissions as described below [22,23]. The AADT and speed data for each link were derived using road counts and travel demand models (TDM) with link-specific inputs including AADT, number of lanes, roadway type and location from the [21], the Michigan Department of Transportation, and the US EPA Office of Transportation and Air Quality. The average speed for each link was estimated for four periods: morning rush hour peak (7–9 AM), mid-day (9 AM–3 PM), afternoon rush hour peak (3 PM–6 PM), and off-peak (6 PM–7 AM).

Hourly traffic flows were derived for each link and vehicle class. The hourly number of vehicles on link i was calculated as:

V_i,k,t_ = FM_NFC(i),k_ MAF_MON(t)_ DAF_k,DAY(t)_ HAF_NFC(i),t_ AADT_i_ (3)

where V_i,k,t_ (counts h^−1^) is the number of vehicles on link i (i = 1 to 9701) for vehicle class k (k = 1 to 8) and hour of the year t (t = 1 to 8760), and AADT_i_ is the annual average daily flow for link i, as noted above. Equation (1) uses three temporal allocation factors to account for variation by month of the year, day of the week, and hour of the day, as well as a fleet mixture factor, each described below. The eight vehicle classes represent aggregations from MOVES emission model and represent motorcycles, light-duty gasoline vehicles, light-duty diesel vehicles, light-duty gasoline trucks with gross vehicle weight (GVW) less than 6001 pounds, light-duty gasoline trucks with GWV > 6001 pounds, light-duty diesel trucks, heavy-duty diesel trucks, heavy-duty gas vehicles, and heavy-duty diesel vehicles (MC, LDGV, LDDV, LDGT1, LDGT2, LDDT, HDGV, HDDV). These classes were derived using state-level data from the Federal Highway Administration, and information from the U.S. EPA Emission Inventory Improvement Program. 

The fleet mix allocation factor FM_NFC(i),k_ (dimensionless) gives the fraction of vehicles in vehicle class k for link i, which depends on its National Functional Class (NFC) designation. Allocation factors were based on Table VM-4 from the FHWA Highway Statistics Series (http://www.fhwa.dot.gov/policyinformation/statistics/2010/vm4.cfm) in conjunction with information from the U.S. EPA Emission Inventory Improvement Program (USEPA, 1996). Modeled NFCs included interstates, other freeways, other principal arterials, minor arterials, major collectors, minor collectors and bridge (NFC designations 11, 12, 14, 16, 17, 19 and 90). For example, for urban interstates (NFC = 11), LDGV and HDDV respectively represent 70.8 and 7.7% of AADT. This adjustment has the constraint that summed across the eight vehicle classes, Σ_k = 1...8_ FM_NFC(i), k_ = 1 for each road link. In Detroit, only three links were designated as NFC = 90, of which one had AADT = 0 and the others were quite short and were 245 and 163 m in length. Since fleet mix allocation factor were not available for NFC = 90, NFC = 11, which had the highest allocation of diesel vehicles, was used.

The month-of-year allocation factor MAF_MON(t)_ (dimensionless, with month indexed by hour t) had values that ranged from 0.86 (December) to 1.10 (August), reflecting higher summer traffic. This allocation factor has the constraint that summed across the 12 months, Σ_t = 1...12_ MAF_t_ = 12.

The day-of-week allocation factor DAF_k,DAY(t_) (dimensionless), where DAY(t) is the day of week (indexed by hour t), has the effect of slightly increasing daily total flows for most vehicle classes on Friday (by 8%), and decreasing flows on Saturday (by 9%) and Sunday (21%), all compared to other weekdays. However, patterns differ for the HDGV and HDDV classes, which have slightly lower flows on Friday (by 3%) and significantly lower flows on Saturday and Sunday (61 and 71%, respectively). This factor has the constraint that summed across the 7 days in a week, Σ_t = 1...7_ DAF_k,DAY(t)_ = 7 for each vehicle class k.

The hour-of-day allocation factor HAF_NFC(i),HR(t),DT(t)_ (dimensionless) represents the proportion of traffic volume for hour of the day (HR(t) = 1 to 24, indexed by hour t) and day type DT(t) = 1 to 3 (indexed by t), respectively representing weekdays, Saturday, and Sunday. This factor was obtained from SMOKE [24]. Separate patterns are used for weekdays, which are typically bimodal with peaks representing morning and afternoon rush hour peaks, and weekends, which are typically unimodal with a broad afternoon peak. However, patterns vary by road type as given by NFC. This factor has the constraint that summed across the 24 h in a day, Σ_t = 1 to 24_ HAF_NFC(i),DT(t),HR(t)_ = 1 for each NFC and DT.

For traffic on holidays, a Sunday schedule was assumed, accomplished by setting both the day-of-week and hour-of-day allocation factors DAF_k,DAY(t_) and HAF_NFC(i),HR(t),DT(t)_ to Sunday values. Holidays in year 2010 considered were New Year’s Day (1 January), Memorial Day (31 May), Independence Day (5 July), Thanksgiving (25 November), and Christmas (25 December).

We confirmed that these adjustments obtained the correct AADT by summing link specific-flows over vehicle classes and hours of the year, that is:

AADTi ≈ 365^−1^ Σ_k = 1...8_ Σ_t = 1to8760_ V_i,k,t_ (4)

Because the AADT does not account for holidays, Equation (4) is not an equality, although the difference between the AADT and the calculated average was very small.

### 2.4. Emissions

A source inventory for NO_x_ and PM_2.5_ was compiled for roads in Detroit and surrounding Wayne County for the year 2010. Hourly estimates of emissions were calculated for each of the 9701 links. First, emission factors for primary exhaust emissions of each pollutant were calculated using MOVES2010a [24]. MOVES is designed to estimate emissions from vehicle sources using a power-based approach. Emission rates in MOVES vary by vehicle class, vehicle speed, ambient temperature, and fuel properties. Thus, emission factors EF_k, SPEED, TEMP, MON_ (g mile^−1^ vehicle^−1^) were calculated for eight vehicle class (k = 1…8), 16 vehicle speeds (2.5, 5, 10, 15 ... 75 mph), 11 ambient temperatures (0, 10, 20 ... 90, 100 °F), and 12 months (January through December). Monthly average properties for fuels in the modeling domain were based on survey information from SEMCOG. MOVES inputs were adjusted for the vehicle age distribution of the 2010 Detroit fleet, based on an analysis of vehicle registration information by the Lake Michigan Air Directors’ Consortium.

The pollutant-specific emission factors from MOVES were applied to each of the 9701 road links in the study domain to generate an hourly and link-by-link emissions inventory that accounted for traffic activity on each link, including the estimated hourly flows of each vehicle type and the average speed for each link and hour. Link-specific emission rates E_i,t_ (g m^−1^ s^−1^) for link i and hour t were calculated as follows:

E_i,t_ = 1.72604 E−07 Σ_k = 1...8_ EF_k, SPEED(i, t), TEMP(t), MON(t)_ V_i,k,t_ (5)

where the first constant converts units of distance (1 mile/1609 m) and time (1 h/3600 s), thus matching the vehicle counts and emission factors from MOVES; EF_k,SPEED(i, t), TEMP(t),Month(h)_ = emission factor (g vehicle^−1^ mile^−1^) from MOVES for link i, vehicle class k, link speed SPEED(i, t), hourly average ambient temperature TEMP(t), and month MON(t); and V_i,k,t_ = number of vehicles per hour for link i, vehicle class k, and hour of the year t, as given in Equation (1). Temperature and vehicle speed were placed into 11 and 16 bins, described earlier, and lookup tables were used to select values. Calculations in Equation (5) were performed for NO_x_ and PM_2.5_. Temperatures in Equation (5) were calculated using the average of five local airport weather stations. This provided a complete and robust dataset. Due to large uncertainties, tire, brake and pavement generated PM is excluded. As mentioned, local neighborhood roads are not included in the road network, but this exclusion will not alter modeling results since these roads contribute a negligible fraction of traffic emissions. Further details on the development of the emissions inventory are provided elsewhere [23].

In summary, the spatially and temporally road network for Detroit contained 9701 links that totaled 3064 km in length. The total PM_2.5_ emissions from all links (the product of the emission rate and link length summed across individual links was 15.9 g s^−1^ or 501 ton yr^−1^. This is comparable to values in a recent inventory for southeast Michigan, which estimated primary on-road PM_2.5_ emissions in Wayne County (a slightly larger area than Detroit) as 1664 ton yr^−1^ in 2008, and projected emissions of 613 tons yr^−1^ in 2018, respectively, a decrease attributable to cleaner vehicles. 

### 2.5. Dispersion Modeling

Primary PM_2.5_ concentrations from vehicle emissions were predicted using RLINE, a steady-state plume-dispersion model [14,25,26] following standard guidance for roadway sources [27]. RLINE incorporates newly developed algorithms for predicting concentrations from on-road sources, e.g., tailpipe emissions from cars, buses, trucks and motorcycles. As a line source model, it uses a numerical integration of multiple point sources along the road link, and it automatically determines the number of points needed to represent each link. Dispersion parameters are derived from field data and recent wind tunnel experiments for near road sources. The model is capable of predicting concentrations at receptors very close to roads, and thus is particularly suited to health and other studies examining near-road concentrations. Unlike other line source models, RLINE simulates the ‘upwind’ concentrations that can result from plume meandering. Currently, RLINE is available as beta test version from US EPA, and it is considered a “research” model (not a “regulatory” model).

### 2.6. Computational Considerations

Estimating annual concentrations over the modeled domain is computationally intensive. Given the 9701 road links, 27,622 receptors, and 8760 h per year, 2.34 trillion source-receptor calculations must be performed for each pollutant. Each source-receptor calculation involves iterative numerical algorithms. For this problem, a standard workstation would require many centuries, and even a large computer cluster can require many months. 

The following steps were used to speed up calculations. First, an analytical solution in RLINE (also incorporated into the beta version of the model) was used. This solution provided similar results to the numerical solution. Second, only link-receptor distances less than 25 km were considered, since roads 25 km or more from a receptor will provide negligible impacts. Third, to limit further the number of source-receptor pairs, an adaptive algorithm accounting for both distance and the magnitude of road link emissions was developed. This algorithm, which shortened the distance cut-off if the emission rate on the link was low, was developed and tested by running RLINE simulations using a medium resolution receptor grid on (worst-case) high pollution days, and comparing results with and without exclusion of link-receptor pairs. Fourth, the spatial resolution and receptor network was adjusted (initially 100 m was considered). Fifth, annual average results were based on a subset of meteorology, specifically, every 6th day in 2010 starting 1/3/2010. The selected 61 days were found to provide representative results (e.g., model runs using a smaller set of receptors and all 365 days of the year gave equivalent concentrations). Sixth, RLINE was recoded to allow variable (hourly) emissions without post-processing, and portions were optimized to eliminate repetitive calculations using lookup tables and other methods. Seventh, the emission generator was revised to eliminate the huge emission inventory files (hourly data for each link), and instead used pre-computed emission profiles for each NFC and speed class combination, an approach that gave identical results. Finally, receptors were broken down into several subsets, and calculations were performed using several computers simultaneously by subset.

These steps described above allowed computation of annual averages at the at 27,622 receptors in approximately 2 days using two workstations simultaneously, with results that were very similar to those that included all link-receptor pairs and the numerical algorithm. For example, PM_2.5_ concentrations were about 0.2 µg/m^3^ lower at most receptors, mainly due to the exclusion of distant sources, and the correlation between streamlined and exact models was 0.984. Agreement was nearly perfect for concentrations above 1.0 µg/m^3^.

## 3. Results and Discussion

Several maps illustrate key modeling results, as described below.

### 3.1. Annual Average PM_2.5_ Concentrations

**Figure 1:**
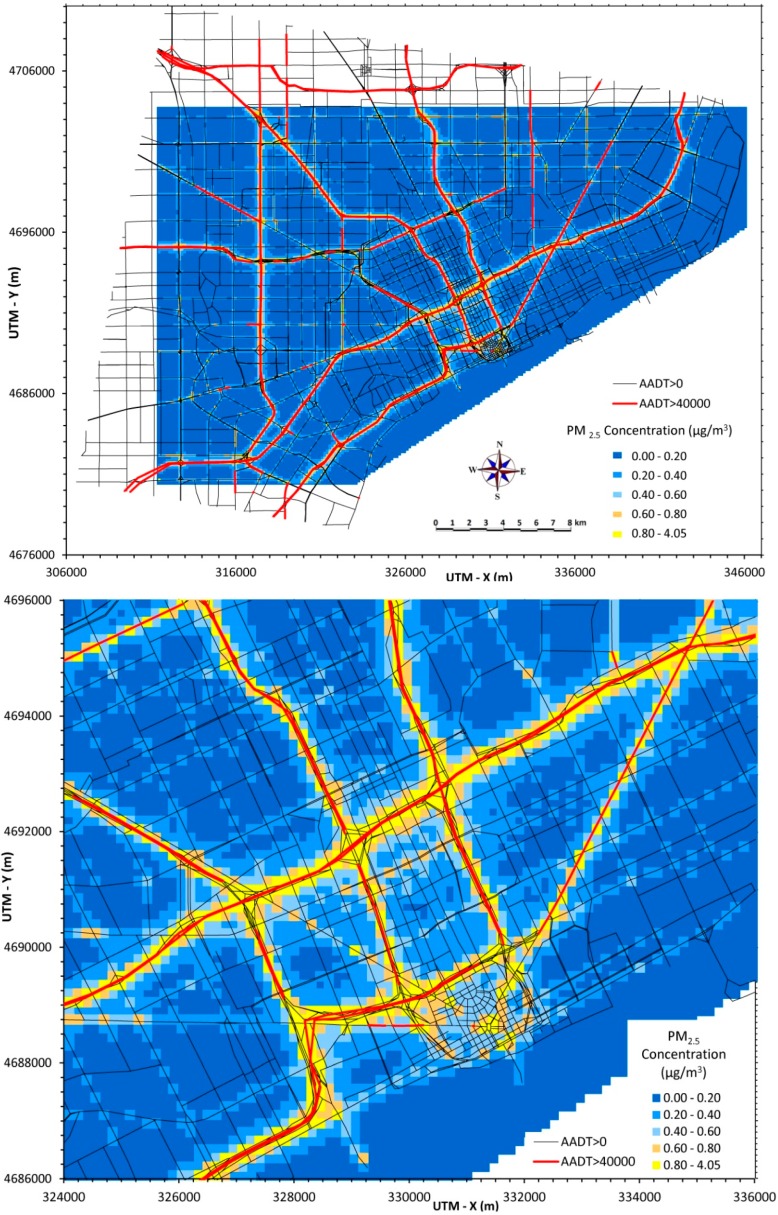
Annual average PM_2.5_ concentrations from traffic emissions across Detroit (40 × 30 km area, top) and in central area (10 × 12 km area, bottom).

Figure 1 (top panel) displays annual average PM_2.5_ levels across the 30 × 40 km Detroit region due to local traffic emissions. The road network extends beyond the receptor network (where concentrations are calculated), which is shown as the shaded area. The lower panel of Figure 1 zooms in on a 10 × 12 km area and shows the detail of the road network and the spatial coverage of each (150 × 150 m) area corresponding to a single receptor. In both panels, X and Y axes use the Universal Transverse Mercator (UTM) projections, and scales are in meters. Each map has grouped concentrations into five levels (shown on the scale in the figures), and each displays results from the annual simulation using hourly data, as described earlier. The highest concentrations occur near major roads, e.g., I-75, I-96, I-94, M-10 and M-39, and particularly at the intersections of these (and other) high traffic roads. Several major arterials also have relatively high concentrations, e.g., 8 Mile Road. The highest annual average concentration is 4.05 µg/m^3^. PM_2.5_ emissions arise from each vehicle class considered, but heavy diesel trucks produce a disproportionate share, thus the highest PM_2.5_ concentrations are near high diesel-traffic roads like I-94 and I-75.

Annual average concentrations might appear to be symmetrical on either side of major roads. However, pollutant levels tend to be higher on the east side of roads, as compared to the west side, an effect of prevailing winds and other meteorological factors. As discussed later, daily averages show much greater variation and asymmetry, e.g., higher concentrations on the downwind side of the road.

Predicted levels of traffic-related air pollutants in Detroit fall below the U.S. national annual ambient air quality standard for PM_2.5_ (currently 12 µg/m^3^ averaged over three years) and below PM_2.5_ levels monitored in Detroit, which currently average around 10 µg/m^3^ (again, Figure 1 displays concentrations from only on-road traffic emissions). Predicted PM_2.5_ levels are comparable to those estimated in recent receptor modeling apportionments in Detroit, which suggest that vehicle emissions contribute 24%–36% of PM_2.5_ at residential sites, with the balance provided by secondary sulfate/coal combustion (17%–35%), secondary nitrate (16%–37%), organic matter (17%–21%), road dust, steel manufacturing, and mixed industrial sources (11%) [28]. Annual average concentrations often are considered the most representative indicator of pollutant levels, particularly when examining effects that depend on long-term exposures, e.g., cancer risk.

### 3.2. Daily Average PM_2.5_ Concentrations

**Figure 2:**
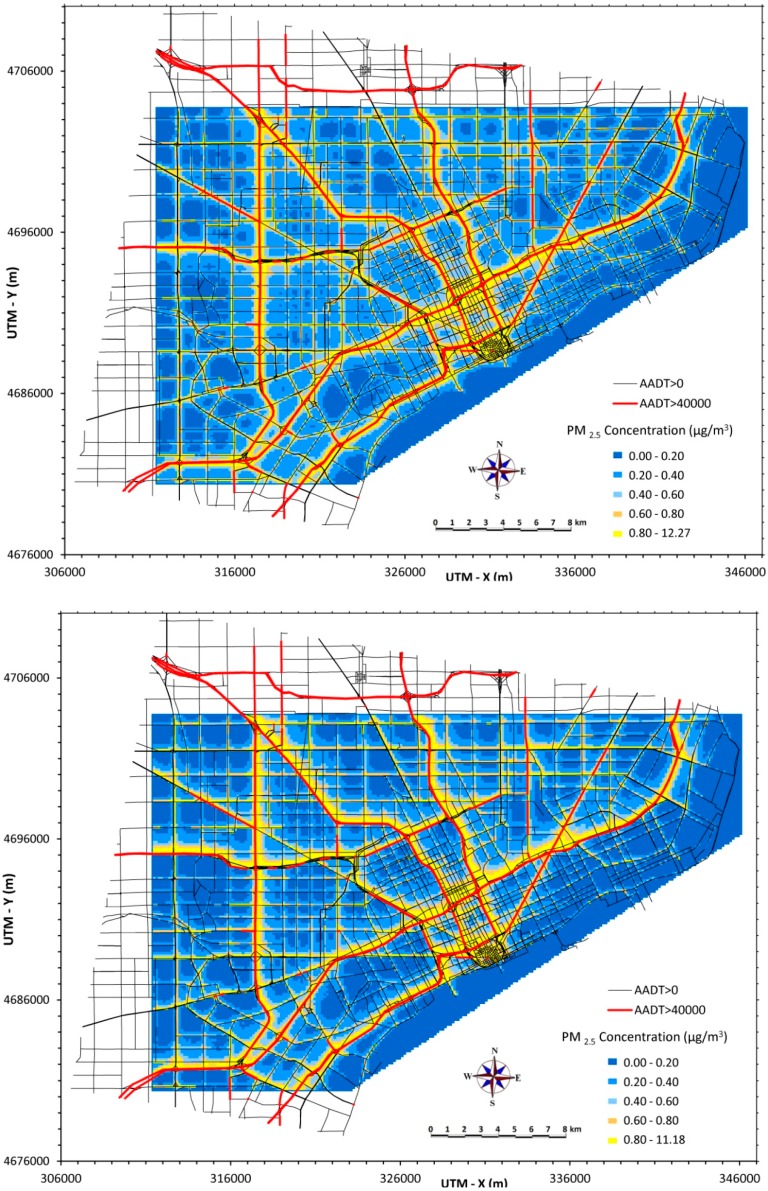
Daily (24-h) PM_2.5_ concentrations from traffic emissions across Detroit (30 × 40 km) for 8 February 2010 (top) and 29 December 2010 (bottom).

Much greater variation is displayed in daily (24-h) averages, as shown in Figure 2, which maps 24-h averages for 8 February 2010 (Monday, top panel) and 29 December 2010 (Wednesday, bottom panel), respectively. These days were selected as two of the higher PM_2.5_ days, a result of poor dispersion conditions and higher emissions (due to weekday traffic and higher emissions during cold temperatures). Concentrations reached 11 to 12 µg/m^3^ at some near-road receptors, and greater asymmetry is observed, especially on 29 December when winds were light (2.2 to 3.3 m s^−1^) and blowing primarily to the north.

### 3.3. Maximum Daily Average PM_2.5_ Concentrations

**Figure 3:**
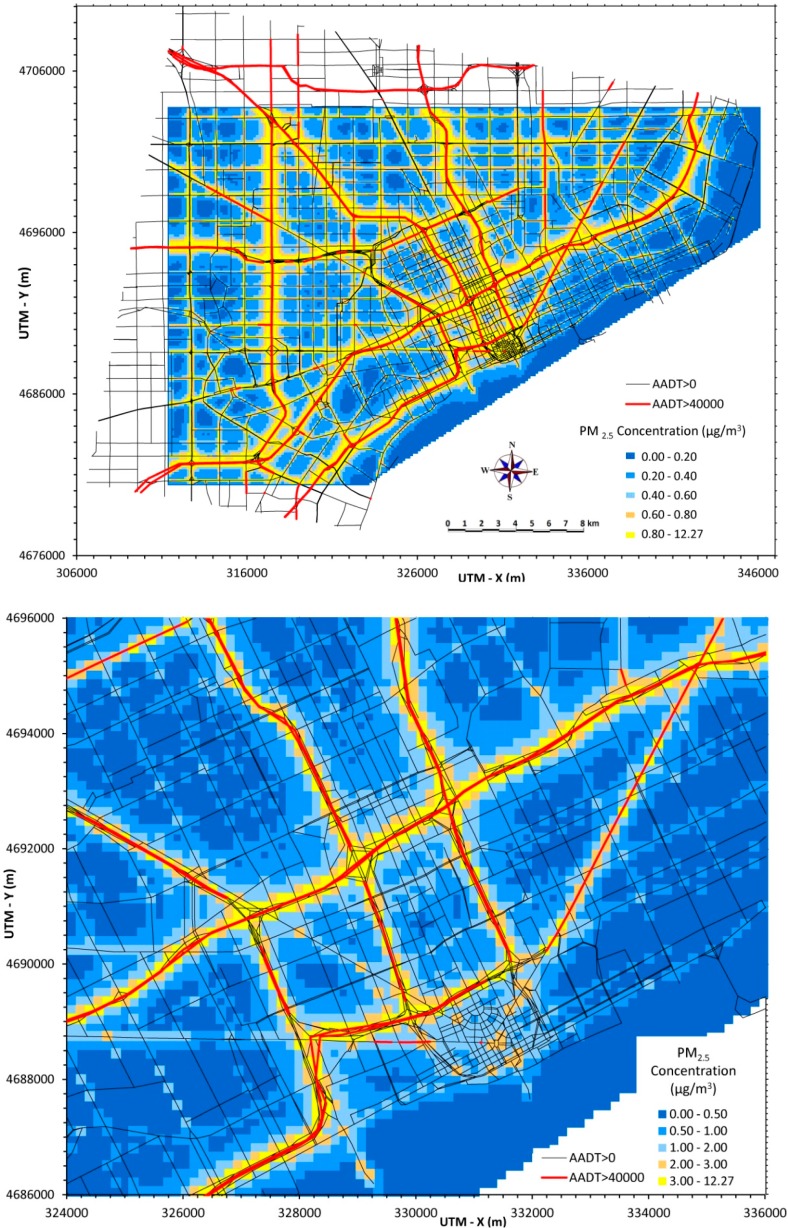
Maximum daily PM_2.5_ concentrations from traffic emissions across Detroit (30 × 40 km area, top) and in central area (10 × 12 km area, bottom).

A third PM_2.5_ example is provided that uses the maximum daily average concentration occurring over the year, determined as the highest 24-h average occurring at each receptor on any day during the year. As shown in Figure 3 (top panel), the portion of Detroit experiencing high concentrations increases with this indicator. 

The maximum daily average PM_2.5_ concentration can be compared to the short-term National Ambient Air Quality Standard, currently 35 µg/m^3^ (98th percentile, averaged over 3 years). Figure 3 (lower panel) shows the maximum daily average for the central portion of Detroit. Note that the concentration scale has been changed to more clearly show gradients. As before, the highest concentrations occur near major roads and intersections. In addition, areas 1 km or more distant from the roadway can experience PM_2.5_ concentrations that are elevated by over 1 µg/m^3^. The maximum daily average concentration is useful to indicate areas that may be affected by high short-term pollutant levels, e.g., potential hotspots, and this indicator is relevant for examining effects that depend on acute exposures, e.g., asthma exacerbation and cardiovascular effects.

### 3.4. Annual Average NO_x_ Concentrations

**Figure 4:**
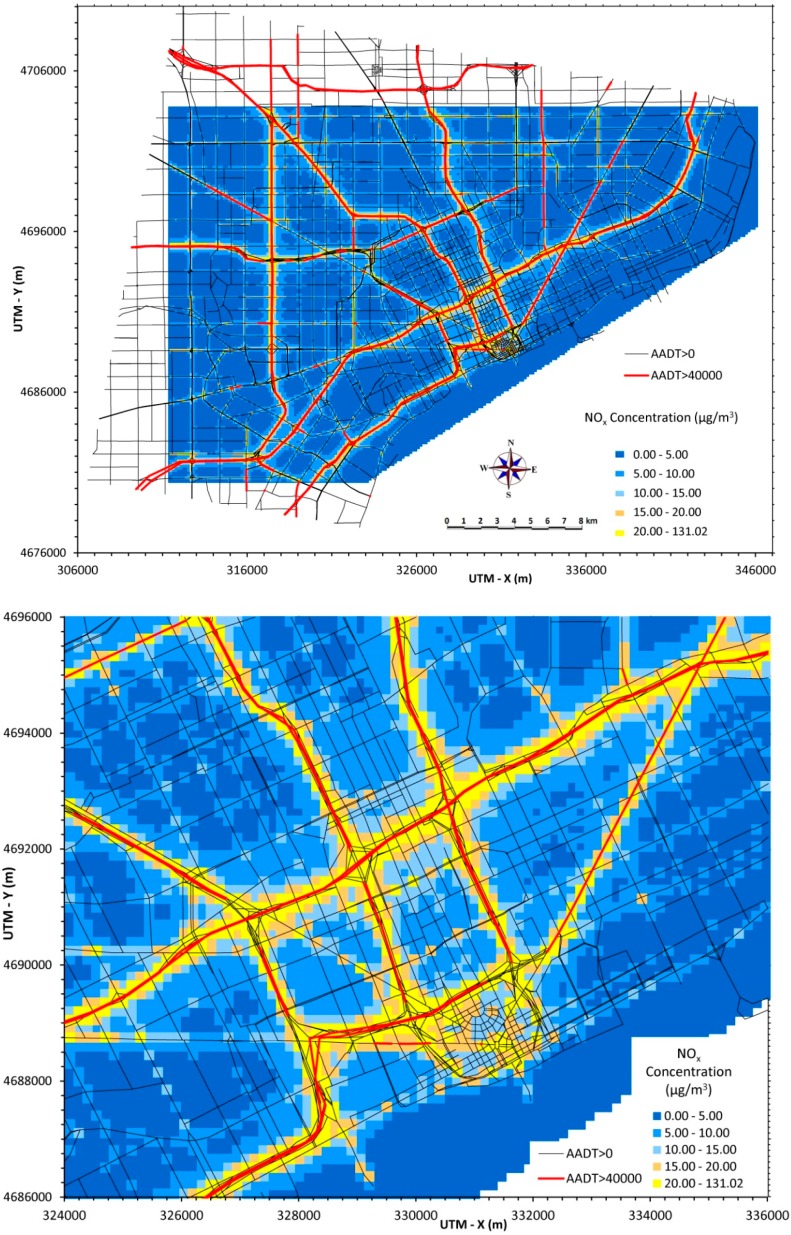
Annual average NO_x_ concentrations from traffic emissions across Detroit (30 × 40 km area, top) and in central area (10 × 12 km area, bottom).

Figure 4 displays annual average predictions of NO_x_ from local traffic emissions for the same regions presented earlier for PM_2.5_ (Figure 1). Vehicle exhaust emissions include both NO and NO_2_, which is summed together as NO_x_. In contrast to PM_2.5_ emissions, which are dominated by diesel-powered vehicles, both gasoline- and diesel-powered vehicles emit substantial levels of NO_x_. Thus, predicted NO_x_ concentrations tend to reflect total traffic (both cars and trucks), thus roads that have extensive car traffic but relatively low truck traffic (e.g., I-96 and M-39) can have NO_x_ levels comparable to roads that have high volumes of both cars and trucks (e.g., I-75 and I-94). Still, NO_x_ and PM_2.5_ concentrations are highly correlated, as shown by the similar spatial patterns in Figure 1 and Figure 4. However, actual concentrations will be affected by background concentrations resulting from non-traffic sources, moreover, chemical transformations producing both secondary PM_2.5_ and NO_2_ will affect concentrations with the effect of decreasing both spatial and temporal correlation. 

Traffic emissions constitute a large and often the dominant source of NO_x_ emissions in urban areas. Analysis of the latest National Emission Inventory data shows that on-road NO_x_ emissions represent 45% of total NO_x_ emissions in Wayne County (encompassing Detroit) and 48% in the seven county southeast Michigan area (http://www.epa.gov/ttn/chief/net/2011inventory.html). Receptor model or other apportionments of NO_x_ are unavailable. Background sources of NO_x_ tend to be less important than for PM_2.5_. To show the importance of vehicle emissions, we simplistically assume that all NO_x_ emissions from vehicles are in the form of NO_2_, *i.e.*, transformation is immediate. In this case, predicted levels approach or exceed the previous U.S. annual average air quality standard (100 µg/m^3^). (Recently, the US NAAQS switched to a 1-h averaging period.) However, the assumption of immediate transformation is not realistic (NO to NO_2_ conversion rates depend on temperature, free radical concentrations and other factors), and NO_2_ levels observed at both near-road and distant sites in Detroit do not exceed either NO_2_ standard. 

### 3.5. Hourly NO_x_ Concentrations with 10 m Resolution

**Figure 5:**
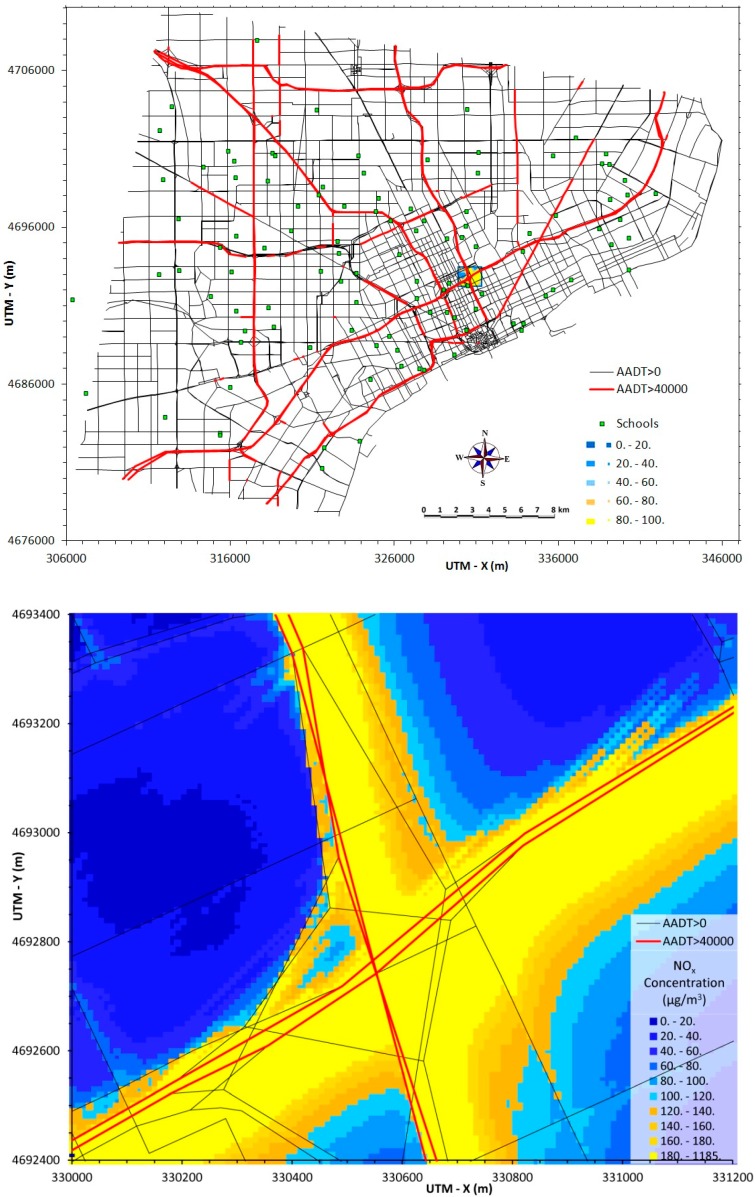
NO_X_ concentrations from traffic emissions for 11 January 2010 at 5 pm for I-75/I-94 area at 10 m resolution. Top: green arrow indicates modeled area; green squares also show locations of schools; Bottom: 1.0 × 1.2 km area modeled.

Figure 5 provides an example of extremely high resolution modeling using receptors on a 10 m grid near a high impact area, the intersection of I-75 and I-94. A 1.0 ×1.2 km region was simulated for the first 12 days in 2012, and the second-highest hour was selected for display. The prevailing meteorology was cold (−6 °C) with low winds (1.1 m/s) from the WNW (314°). NO_x_ concentrations show smooth gradients, large decreases in concentrations at a distance of 200 to 300 m from the major roads, and results that generally match those seen in the earlier figures. Such modeling might be used to evaluate compliance with the 1-hour ambient air quality standard for NO_2_. 

Generally, it is impractical to model large areas with very fine resolutions like 10 m. For example, compared to the 150 m receptor network, the 10 m network requires 225 times more receptors (e.g., 7 million receptors for Detroit). Results obtained at 150 m resolution may adequately represent concentrations at locations that are close (e.g., within 50 to 75 m) to major roads. As often used in modeling point sources, “nested” sets of receptors might be used, e.g., a 10 or 20 m grid might be used very near major roads, a 100 or 150 m grid at intermediate distances, and a coarser grid at longer distances.

### 3.6. Applications

The use of geocoded data and geographical information systems (GIS) has become routine in many types of environmental analyses. Surrogates of pollutant exposure have been widely used, such as the distance from residences or schools to highways or Superfund sites [18,19]. However, surrogates can have significant limitations: they incompletely or improperly account for the nature of emission sources, effects of meteorology, orographic features, small scale variation in pollutant concentrations, time-activity patterns of emissions and the study subjects, and other factors that can affect pollutant emissions, transport, fate and exposure. In consequence, results may be biased and exposures may be misclassified [12]. Another important limitation is that quantitative exposure estimates are not obtained, which limits interpretations and use in policy development and management since results cannot be compared to ambient air quality standards. 

Several recommendations are made for using spatially- and temporally-resolved air quality information. First, to obtain population-specific exposures, this information can be mapped to census block groups, census blocks and parcels. Mapping to the smaller geographic units is preferable since larger units such as zip code and block groups do not provide the necessary spatial resolution to provide accurate results [29]. Second, as noted in the Introduction, spatially-and temporally-resolved data can be utilized in epidemiology studies that link exposures and health outcomes, e.g., cardiovascular disease, asthma symptoms, and viral infections. Ideally, this requires the collection of health surveillance data on a spatial scale that matches the sharp gradients demonstrated in this paper for traffic related air pollutant concentrations. To reflect representative or typical levels, the use of annual average data is preferred. To show potential hotspots, the use of the maximum daily average is preferred. Third, air quality data can identify pollutant “hotspots” and critical areas with potentially vulnerable populations, e.g., schools, hospitals, parks, athletic fields and other locations where children and other susceptible individuals may be exposed. For example, air quality data could be incorporated into analyses assessing the impacts of highways on schools [18]. 

The development and evaluation of policies relevant to traffic-related air pollutants can benefit from spatially explicit pollutant data. This includes the development of recommendations or guidelines to reduce exposure and adverse health impacts using buffers (e.g., vegetated linear “parks”) along highways [30], minimum separation distances for schools and other critical facilities from highways, and requirements for filters, air intakes and other ventilation system controls in buildings near major roads [31]. The relevance of environmental impact assessments and other analyses conducted for transportation projects, including corridor and transit-oriented development, would be enhanced by incorporating spatially resolved analyses of pollutants. In addition, such data could improve estimates regarding the environmental and health benefits of emission reduction policies, such as retrofits to older buses and trucks, improved transit options, and incentives for zero-emission vehicles. 

Additional applications of highly resolved air quality information pertain to forecasts of future air quality and associated impacts. At the project-level, spatially-resolved predictions of future air quality could be used to ensure compliance with standards and to evaluate exposure and environmental justice implications of specific actions, e.g., the environmental impact assessment for a proposed freeway expansions might examine impacts using the spatially-resolved predictions. In this case, emissions would use projected rather than historical vehicle volumes. More broadly, predictions can be used in exposure, impact, risk and health assessment studies to quantify exposure, estimate risks, and determine the fraction of attributable disease. As an example, health impact studies might incorporate Census and surveillance data to identify the numbers of individuals exposed to specific pollutant levels, adjust for time activity patterns (e.g., time outdoors) and other factors (e.g., prevalence of existing disease), and then predict the expected mortality and morbidity impacts using concentration-response functions [32,33]. Such predictions can help evaluate the health consequences of alternative mitigation options aimed at reducing emissions and exposures, e.g., use of optimized traffic signaling, improved emission controls, and buffers around highways or critical facilities (e.g., schools and playgrounds). Lastly, predictions of air quality and its sequel are useful for the development of yet more comprehensive “sustainability indicators.” These might overlay air pollutant data with other environmental, social and physical stressors, e.g., household income, access to medical facilities, housing quality, noise, *etc.*

### 3.7. Limitations

Like any modeling study, the limitations and uncertainties of predictions should be recognized. Simulation modeling involves a large number of parameters, assumptions, and input data. While predictions use the best available information, uncertainties can be large and actual concentrations will likely differ from predictions. For example, hills, tall buildings and other obstacles can modify the wind field and affect predictions, particularly short term maximum concentrations. The practical ability to model urban-scale micrometeorological phenomenon is limited, and airport meteorological data often are considered to be representative. This applies to Detroit where topography is flat and buildings (outside a small urban core) are mostly low-rise. As a second example, only primary pollutants are modeled, thus neither NO_2_, nitrate (both resulting from NO_x_ emissions) or secondary organic aerosol is predicted. Despite such limitations, the approach used is believed to represent actual conditions better than previous models, a result of RLINE’s tailoring for near-road environments and the extensive road network used. Errors will likely decrease at longer averaging times, e.g., annual average estimates will likely be more reliable than estimates for a particular hour or day. While absolute levels of predictions involve uncertainties, spatial patterns are likely to be accurate. Additional spatial resolution, as shown with the 10 m grid, would likely increase the highest concentrations since more receptors would be placed very close to roads. However, such fine resolution also brings into play issues related to the accuracy of geocoding of roads and other features [34], the increased computational burden discussed earlier, and fundamental issues regarding the formulation of plume models. 

This paper presents results that include contributions from only on-road traffic exhaust emissions. As discussed, predictions excluded stationary sources (e.g., power plants, boilers) and distant or “background” sources (located out of the study area). Only primary emissions from traffic-related sources were modeled (chemical reactions that were not modeled), and PM_2.5_ derived from road, tire and brake wear was not modeled. The available data and source apportionments suggest that the predictions are in a reasonable range; however, no attempt was made to validate the model in this paper. The hybrid modeling system used in Detroit for both mobile, stationary and background sources has shown reasonably good agreement to observations obtained at fixed monitoring sites [26]. The use of site-specific time allocation factors for vehicle activity may improve model performance, especially for short-term averages [35]. Only one year (2010) was evaluated. Generally, very similar results are obtained using meteorology for other years, but changes in traffic and emissions rates over the years may alter results. In particular, on-road diesel emissions of both PM_2.5_ and NO_x_ have been greatly reduced, a result of the use of low sulfur fuels and emission controls that were phased in starting around 2007.

## 4. Conclusions

This study has shown the level and spatial distribution of PM_2.5_ and NO_x_ concentrations in Detroit attributable to on-road emissions. The modeling involved a uniquely high degree of temporal and spatial resolution that allows investigation of short- and long-term pollutant impacts and near-road concentrations. On-road emissions are significant given the extent of commuting, extensive truck traffic, the international crossing, and the many houses, schools and other populated areas located near major roads where pollutant concentrations and exposures are often highest. The spatial patterns for annual averages and high pollution days show contrasting patterns, with the highest concentrations occurring near major roads and intersections of major roads. Understanding the distribution of pollutant concentrations along near-road environments in urban areas such as Detroit can be particularly important since many residents may be medically underserved and disproportionately suffer from diseases linked to environmental factors. 

High resolution pollutant data are essential for many types of analyses. These include identification of pollutant “hotspots”, “project-level” analyses of future transportation options, use as exposure measures in epidemiology studies, delineation of populations that are vulnerable and susceptible (e.g., children and elderly at schools, hospitals, parks, and athletic fields), and policy-level analyses examining risks and benefits of mitigation options and transportation improvements. We also suggest their use in composite “sustainability” or “vulnerability” indicators developed by overlaying air pollutant data with spatial information representing other environmental, social, or health stressors. Spatially-resolved pollutant data increase the relevance and potentially the accuracy of such applications.
